# Red Organic Light‐Emitting Diode with External Quantum Efficiency beyond 20% Based on a Novel Thermally Activated Delayed Fluorescence Emitter

**DOI:** 10.1002/advs.201800436

**Published:** 2018-07-20

**Authors:** Jia‐Xiong Chen, Kai Wang, Cai‐Jun Zheng, Ming Zhang, Yi‐Zhong Shi, Si‐Lu Tao, Hui Lin, Wei Liu, Wen‐Wen Tao, Xue‐Mei Ou, Xiao‐Hong Zhang

**Affiliations:** ^1^ Institute of Functional Nano & Soft Materials (FUNSOM) and Jiangsu Key Laboratory for Carbon‐Based Functional Materials & Devices Soochow University Suzhou Jiangsu 215123 P. R. China; ^2^ School of Optoelectronic Science and Engineering University of Electronic Science and Technology of China (UESTC) Chengdu Sichuan 610054 P. R. China

**Keywords:** high external quantum efficiency, red organic light‐emitting diodes, rigid segments, thermally activated delayed fluorescence

## Abstract

A novel thermally activated delayed fluorescence (TADF) emitter 12,15‐di(10*H*‐phenoxazin‐10‐yl)dibenzo[*a*,*c*]dipyrido[3,2‐*h*:2′,3′‐*j*]phenazine (DPXZ‐BPPZ) is developed for a highly efficient red organic light‐emitting diode (OLED). With rigid and planar constituent groups and evident steric hindrance between electron‐donor (D) and electron‐acceptor (A) segments, DPXZ‐BPPZ realizes extremely high rigidity to suppress the internal conversion process. Meanwhile, the highly twisted structure between D and A segments will also lead to an extremely small singlet–triplet energy split to DPXZ‐BPPZ. Therefore, DPXZ‐BPPZ successfully realizes an efficient fluorescent radiation transition and reverse intersystem crossing process, and possesses an extremely high photoluminescence quantum efficiency of 97.1 ± 1.1% under oxygen‐free conditions. The OLED based on DPXZ‐BPPZ shows red emission with a peak at 612 nm and a Commission Internationale de L'Eclairage (CIE) coordinate of (0.60, 0.40), and it achieves high maximum forward‐viewing efficiencies of 20.1 ± 0.2% (external quantum efficiency), 30.2 ± 0.6 cd A^−1^ (current efficiency), and 30.9 ± 1.3 lm W^−1^ (power efficiency). The prepared OLED has the best performance among the reported red TADF OLEDs. These results prove that DPXZ‐BPPZ is an ideal candidate for red TADF emitters, and the designing approach is valuable for highly efficient red TADF emitters.

Since highly efficient thermally activated delayed fluorescence (TADF) emitters were introduced into organic light‐emitting diodes (OLEDs), TADF‐based OLEDs have attracted great attention by their promising full exciton utilization and are considered as the third generation of OLEDs.^1^, ^2^, ^3^, ^4^ Generally, efficient reverse intersystem crossing (RISC) process and high fluorescence quantum yield (*Φ*_F_) are two essential requirements for efficient TADF emitters.^1^, ^5^ For the former one, an extremely small singlet‐triplet energy split (Δ*E*_ST_) between lowest singlet excited state (S_1_) and lowest triplet excited state (T_1_) is highly desired to up‐convert triplet excitons to singlet excitons through thermal excitation.^4^, ^6^, ^7^, ^8^, ^9^, ^10^ Thus, electron‐donor (D)–electron‐acceptor (A) frameworks with a highly twisted way are generally employed to construct TADF emitters, as they can well isolate highest occupied molecular orbitals (HOMOs) in D moieties and lowest unoccupied molecular orbitals (LUMOs) in A moieties, respectively, resulting in small Δ*E*_ST_s and thus effective RISC process.^11^, ^12^, ^13^, ^14^, ^15^ While for the latter requirement, *Φ*_F_ is determined by the following equation^16^, ^17^

(1) $$\Phi_{F}=\;\frac{k_{F}}{k_{F}+\;k_{\mathrm{IC}}+k_{\mathrm{ISC}}}$$

where *k*_F_ is the rate constant of fluorescence decay, *k*_ISC_ is the rate constant of intersystem crossing (ISC) process from S_1_ to T_1_ state, and *k*_IC_ is the rate constant of internal conversion (IC) process from S_1_ to ground state (S_0_). According to Equation (1), both ISC and IC processes are against the fluorescence process. However, only the IC process results in a direct exciton loss, because triplet excitons generated from ISC process can be reutilized through the RISC process with enough efficiency.^18^, ^19^ Thus, the most fundamental point for *Φ*_F_ is suppressing the influence of IC process from S_1_ to S_0_ state. For aromatic compounds, *k*_IC_ is approximately decided by the following equation^20^

(2) $$k_{\mathrm{IC}}=\;10^{13}e^{-\alpha \;\Delta \;E}$$

where Δ*E* is the energy gap between S_1_ and S_0_ state and α is a proportionality constant decided by molecular rigidity. According to Equation (2), *k*_IC_ is sensitive to Δ*E* and exponentially magnified with the Δ*E* decreased. For the emitters with high Δ*E* values (i.e., blue and green emitters), the nonradiative transitions of singlet excitons are naturally suppressed according to Equation (2), and *k*_IC_ is far lower than *k*_F_ so that *k*_IC_ can be generally ignored.^16^ Thus, efficient RISC process and high *Ф*_F_ can be easily realized for blue and green TADF emitters. In the past several years, great progresses have been made in blue and green TADF‐based OLEDs, approaching or even exceeding the external quantum efficiencies (EQEs) of 20%.^21^, ^22^, ^23^, ^24^ However, with the decreasing of Δ*E* of TADF emitters into the long‐wavelength area (i.e., red emitters), significantly increased *k*_IC_s would become comparable with or even higher than *k*_F_s.^25^, ^26^, ^27^ Thus, as shown in **Figure** **1**a, when developing red TADF emitters based on the design strategy of high‐performance blue and green TADF emitters, the IC process would play an evident role in energy transfer diagrams, leading to terrible energy loss. For example, the reported red TADF molecule AQ‐DMAC (a4, Figure S1a, Supporting Information) constructed with a conventional highly twisted D–A structure for TADF emitters^17^ shows an extremely small Δ*E*_ST_ of 0.08 eV. But its *k*_IC_ is over 10 times higher than *k*_F_, thus AQ‐DMAC cannot used as an emitter.

**Figure 1:**
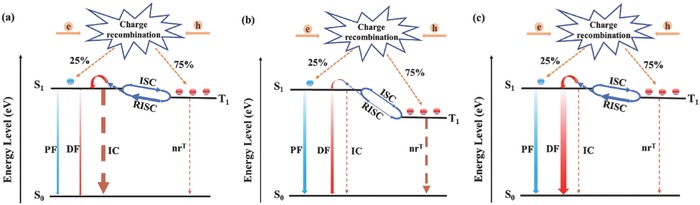
Energy transfer diagrams of red TADF emitters. PF: prompt fluorescence; DF: delayed fluorescence; IC: internal conversion; nr^T^: nonradiative decay of triplet excitons; ISC: intersystem crossing; RISC: reverse intersystem crossing. a) Conventional D–A structure: the main triplet exciton transfer channel is RISC process and the main singlet decay channel is IC process. b) Increasing *Φ*_PL_ but suffering large Δ*E*_ST_: the main triplet exciton transfer channel is nr^T^ process and the main singlet decay channel is fluorescence process (including PF and DF). c) DPXZ‐BPPZ: the main triplet exciton transfer channel is RISC process and the main singlet decay channel is fluorescence process (including PF and DF).

To develop efficient red TADF emitters, in 2014, Adachi and co‐workers^17^ proposed that increasing the *k*_F_ via enhancing the conjugation between D and A segments can reduce the influence of *k*_IC_ and used an unconventional D‐phenyl‐A (D‐Ph‐A) structure to develop red TADF emitters (Figure 1b). Based on this method, they reported a TADF red emitter AQ‐TPA (b1, Figure S1b, Supporting Information), whose *k*_IC_ is less than a quarter of *k*_F_. And the OLED based on AQ‐TPA exhibits a maximum EQE of 12.5%.^17^ They also reported a red TADF emitter HAP‐3TPA (Figure S1c, Supporting Information) using the same method.^27^ And HAP‐3TPA exhibits a high maximum EQE of 17.5% in the device, which is the highest result among the red TADF‐based OLEDs till now. Similarly, in 2017, Wang and co‐workers reported a series of long‐wavelength TADF emitters, in which, the D‐Ph‐A structure red TADF emitters also achieved better device performance than the D–A structure ones. The red device using DPA‐Ph‐DCPP (Figure S1d, Supporting Information) as the emitter shows a maximum EQE of 15.1%.^26^ However, with the enhanced conjugation between D and A segments, all these molecules suffer large Δ*E*_ST_s higher than 0.17 eV, which weaken the RISC process and lead to the energy loss from triplet excitons.^28^, ^29^ The efficiencies of these red TADF‐based OLEDs are evidently lower than the theoretical ceiling of 20% EQE.^30^, ^31^ Further development of red TADF‐based OLEDs is highly desired.

To really achieve the full exciton utilization, in this work, we designed and synthesized a novel red TADF emitter, 12,15‐di(10*H*‐phenoxazin‐10‐yl)dibenzo[*a*,*c*]dipyrido[3,2‐*h*:2′,3′‐*j*]phenazine (DPXZ‐BPPZ). As shown in **Figure** **2**, DPXZ‐BPPZ is constructed with a conventional D–A structure by using dibenzo[*a*,*c*]dipyrido[3,2‐*h*:2′,3′‐*j*]phenazine (BPPZ) as the A segment and phenoxazine (PXZ) as the D segment. Compared with the A segments used in reported red TADF emitters,^17^, ^25^, ^26^, ^27^ BPPZ possesses a significantly larger, completely planar and fully conjugated structure without any branched group, thus, it can evidently suppress harmful structural relaxation. On the other side, electron‐donating PXZ does not only exhibit suitable HOMO energy level to realize red emission cooperating with BPPZ, but is also more rigid than other D segments, like diphenylamine (DPA) and 9,9‐dimethyl‐9,10‐dihydroacridine (DMAC). By directly linking one BPPZ with two PXZ, DPXZ‐BPPZ will realize extremely high rigidity due to its rigid and planar constituent groups and evident steric hindrance between D and A segments, which will realize a significantly larger α and suppress the influence of IC process according to Equation (2). Meanwhile, evident steric hindrance between PXZ and BPPZ segments will also lead a highly twisted structure to DPXZ‐BPPZ, which can induce a small Δ*E*_ST_ for efficient RISC process. Thus, DPXZ‐BPPZ has the potential to simultaneously achieve a high *Ф*_F_ and an effective RISC process. As shown in Figure 1c, DPXZ‐BPPZ may possess more effective energy transfer routes than currently reported red TADF emitters. It would have negligible energy loss through the nonradiative processes and achieve a nearly full exciton utilization in the OLEDs. As expected, DPXZ‐BPPZ successfully exhibits an extremely small Δ*E*_ST_ of 0.03 eV as well as a high *k*_F_ value of almost 33 times higher than *k*_IC_, resulting in possesses an extremely high photoluminescence (PL) quantum efficiency (*Φ*_PL_) of 97.1 ± 1.1% under oxygen‐free condition. In the device, DPXZ‐BPPZ exhibits a red emission with a peak at 612 nm and an excellent performance of a maximum forward‐viewing EQE of 20.1 ± 0.2% without any light out‐coupling enhancement. To the best of our knowledge, this is the first time that red TADF‐based OLED achieves a forward‐viewing EQE exceeding 20%. Moreover, the device exhibits relatively low efficiency roll‐off, EQEs remaining 16.7 ± 0.3% at 1000 cd m^−2^ and 12.2 ± 0.3% at 5000 cd m^−2^. These results not only prove that DPXZ‐BPPZ can be an ideal candidate for fabrication of TADF‐based red OLEDs, but also present a successful example of using rigid and aromatic planar segments to design highly efficient red TADF emitters.

**Figure 2:**
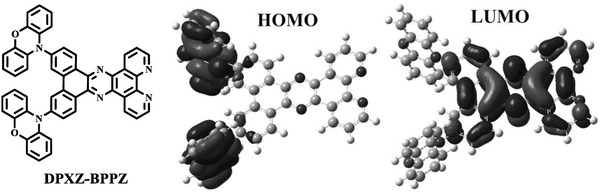
Molecular structure of DPXZ‐BPPZ and its calculated spatial distributions of the HOMO and LUMO.

The synthetic process of the designed molecule DPXZ‐BPPZ is shown in Scheme S1. DPXZ‐BPPZ is synthesized via a cyclization process by adding 1,10‐phenanthroline‐5,6‐diamine to 3,6‐di(10H‐phenoxazin‐10‐yl)phenanthrene‐9,10‐dione, which is obtained using Buchwald–Hartwig crosscoupling reaction from 3,6‐dibromophenanthrene‐9,10‐dione and PXZ. The structure of DPXZ‐BPPZ is confirmed by mass spectroscopy (MS) and nuclear magnetic resonance (NMR) spectroscopy.

Theoretical calculations were first performed to predict the molecular characteristics of DPXZ‐BPPZ. Figure 2 shows the frontier orbital distributions at the optimized geometry. The HOMO is well isolated on both of PXZ segments, and LUMO is mainly localized on BPPZ core, resulting in a well frontier orbital separation. Moreover, optimized S_1_ and T_1_ states of DPXZ‐BPPZ were also studied by using time‐dependent density functional theory (TD‐DFT) calculation. As shown in Figure S2a in the Supporting Information, in the natural transition orbitals at S_1_ state, “hole” and “particle” are well separated in PXZ and BPPZ moieties, respectively. Meanwhile, from its spin‐density distribution at T_1_ state (Figure S2b, Supporting Information), spin density is distributed among almost the whole molecule. These results prove its strong intermolecular charge transfer (ICT) characters in both S_1_ and T_1_ states. As expected, an extremely small Δ*E*_ST_ of 0.01 eV is theoretically calculated, and the low Δ*E*_ST_ would lead to an effective RISC process and benefit the utilizations of triplet excitons. In our latest report, PXZ is known as a “pseudoplanar” group, and its dual conformations will lead two possible conformations (the nearly orthogonal and the nearly planar conformations) to PXZ‐based D–A molecules. Particularly, the nearly planar conformation may cause the triplet exciton loss.^32^ Thus, the distributions of two possible conformers were investigated for DPXZ‐BPPZ. As shown in Figure S3 in the Supporting Information, from the potential energy surface of DPXZ‐BPPZ at the ground state, dual valleys are found at the dihedrals between D and A segments at around 0° and 90°, respectively. And the nearly orthogonal conformation, which is further optimized at a dihedral of 80°, is the most stable conformation due to the strong steric hindrances and weak interactions between PXZ segments and BPPZ core. Thus, the nearly orthogonal molecule of DPXZ‐BPPZ is expected to have a high population, and the influence of nearly planar conformation can be ignored.

To confirm the high rigidity and excellent thermal stability of DPXZ‐BPPZ, thermal properties were measured by differential scanning calorimetry and thermogravimetric analysis (TGA) under nitrogen atmosphere. During the whole measurement, no clear glass transition was observed for DPXZ‐BPPZ, which is consistent with its high rigidity. The decomposition temperature (*T*_d_, corresponding to 5% weight loss) is estimated to be as high as 482 °C (Figure S4, Supporting Information), indicating DPXZ‐BPPZ has high thermal stability and is suitable for vacuum deposition.

The ultraviolet–visible (UV–vis) absorption and emission spectra of DPXZ‐BPPZ were measured under different conditions to study its photophysical properties. As shown in **Figure** **3**a, in dilute toluene solution, DPXZ‐BPPZ exhibits a broad weak absorption in the range of 420–540 nm, which could be attributed to ICT transition from PXZ segments to BPPZ core. And the sharp and stronger absorptions around 400 and 320 nm can be assigned to the local excited π–π* transitions of BPPZ and PXZ segments, respectively. In dilute toluene solution at room temperature, DPXZ‐BPPZ shows a weak red fluorescence with a peak at 610 nm. The weak fluorescence and evident Stokes shift should be ascribed to a strong fluorescence quenching effect between DPXZ‐BPPZ and polar solvent molecules. Thus, we further measured its fluorescence spectrum at 77 K to suppress this quenching and molecular‐free relaxations. As shown in Figure 3b, the emission spectrum exhibits a large hypsochromic shift with a peak at 550 nm in toluene. By changing the solvent from the lower polar toluene to higher polar solvent, the emission peak of DPXZ‐BPPZ at 77 K exhibits a significantly red‐shift from 550 nm in toluene to 576, 586, and 605 nm in dichloromethane (DCM), ethyl acetate (EtOAc), and tetrahydrofuran (THF), respectively. The significant solvatochromic effect suggests the strong ICT characteristic of DPXZ‐BPPZ. From the onsets of the fluorescence and phosphorescence spectra of 18 wt% DPXZ‐BPPZ doped in 4,4′‐di(9*H*‐carbazol‐9‐yl)‐1,1′‐biphenyl (CBP) film (Figure 3c), the S_1_ and T_1_ energy levels of DPXZ‐BPPZ are estimated to be 2.41 and 2.38 eV, respectively, and thus its Δ*E*_ST_ is calculated to be 0.03 eV. With such an extremely small Δ*E*_ST_, an effective RISC process can be expected and will result in a highly efficient triplet exciton utilization. The transient PL decay characterization was measured to prove the TADF characteristic of DPXZ‐BPPZ. At room temperature, a prompt decay exhibited a lifetime (τ_p_) of 33 ns in the range of 200 ns (Figure S5, Supporting Information), which is attributed to the direct fluorescence process from S_1_ to S_0_. Meanwhile, in nitrogen atmosphere, a double‐component emission decay profile was obtained in the time range of 400 µs with a delayed lifetime (τ_d_) of 10.3 µs. This result is significantly shorter than the most of reported red TADF emitters,^17^, ^26^, ^27^ suggesting a more efficient RISC process can be expected. In addition, as shown in Figure 3d, with the temperature increasing from 100 to 300 K, the delayed lifetime of DPXZ‐BPPZ is gradually declined, which is consistent with its TADF characteristic. By using an integrating sphere, the absolute *Φ*_PL_ of 18 wt% DPXZ‐BPPZ‐doped CBP film was measured to be an extremely high value of 97.1 ± 1.1% under oxygen‐free condition, which is the highest one among the reported red TADF emitters.^15^, ^17^, ^25^, ^26^, ^27^, ^33^ Therefore, according to Equation (1), the *k*_F_ value is almost 33 times larger than that of *k*_IC_ for DPXZ‐BPPZ, resulting in an negligible IC process contribution to exciton loss. Thus, we assumed all exciton loss on DPXZ‐BPPZ is via the nonradiative transitions of triplet excitons. And the main kinetic parameters of DPXZ‐BPPZ can be estimated according to the following equations^1^, ^16^, ^17^, ^18^

(3) $$k_{p}=\;\frac{1}{\tau_{p}}$$

(4) $$k_{F}\;=\;k_{p}\Phi_{F}$$

(5) $$k_{p}\;=k_{F}\;+k_{\mathrm{ISC}}$$

(6) $$k_{\mathrm{TADF}}=\;\frac{\Phi_{\mathrm{TADF}}}{\;\Phi_{\mathrm{ISC}}\tau_{\mathrm{TADF}}}$$

(7) $$k_{\mathrm{RISC}}=\frac{k_{p}k_{\mathrm{TADF}}}{k_{\mathrm{ISC}}}\;\frac{\Phi_{\mathrm{TADF}}}{\Phi_{F}}$$

(8) $$k_{\mathrm{nr}}^{T}=k_{\mathrm{TADF}}\;-\left(1-\frac{k_{\mathrm{ISC}}}{k_{F}+k_{\mathrm{ISC}}}\right)k_{\mathrm{RISC}}$$

where *Φ*_TADF_ is the delayed fluorescence quantum efficiency; *Φ*_ISC_ is the ISC efficiency; *k*_p_, *k*_RISC_, and *k*_TADF_ are the rate constants of prompt fluorescence, the RISC, and delayed fluorescence decay, respectively; $k_{\mathrm{nr}}^{T}$ is the nonradiative decay rate constant of triplet excitons. *Φ*_F_ and *Φ*_TADF_ are estimated from the *Φ*_PL_ with a relative ratio of 2:3 which was calculated from the transient PL spectra according to the literature.^34^ Therefore, the *k*_F_ and *k*_ISC_ values were estimated to be 1.18 × 10^7^ and 1.85 × 10^7^ s^−1^, respectively. And the *k*_RISC_, *k*_TADF_, and $k_{\mathrm{nr}}^{T}$ are respectively estimated to be 2.24 × 10^5^, 9.23 × 10^4^, and 0.5 × 10^4^ s^−1^. As detailed shown in Table S2 in the Supporting Information, compared with currently reported red TADF emitters, DPXZ‐BPPZ possesses the lowest *k*_IC_, highest *k*_TADF_, as well as a comparative *k*_F_, suggesting its superiority. Moreover, for DPXZ‐BPPZ, the *k*_RISC_ value is 44.8 times higher than $k_{\mathrm{nr}}^{T}$, which indicates nonradiative decay of triplet excitons can be also nearly ignored compared with the efficient RISC process. Based on these negligible *k*_IC_ and $k_{\mathrm{nr}}^{T}$, DPXZ‐BPPZ can indeed achieve the energy transfer diagram as shown in Figure 1c, and it is expected to have full exciton utilization in the OLEDs.

**Figure 3:**
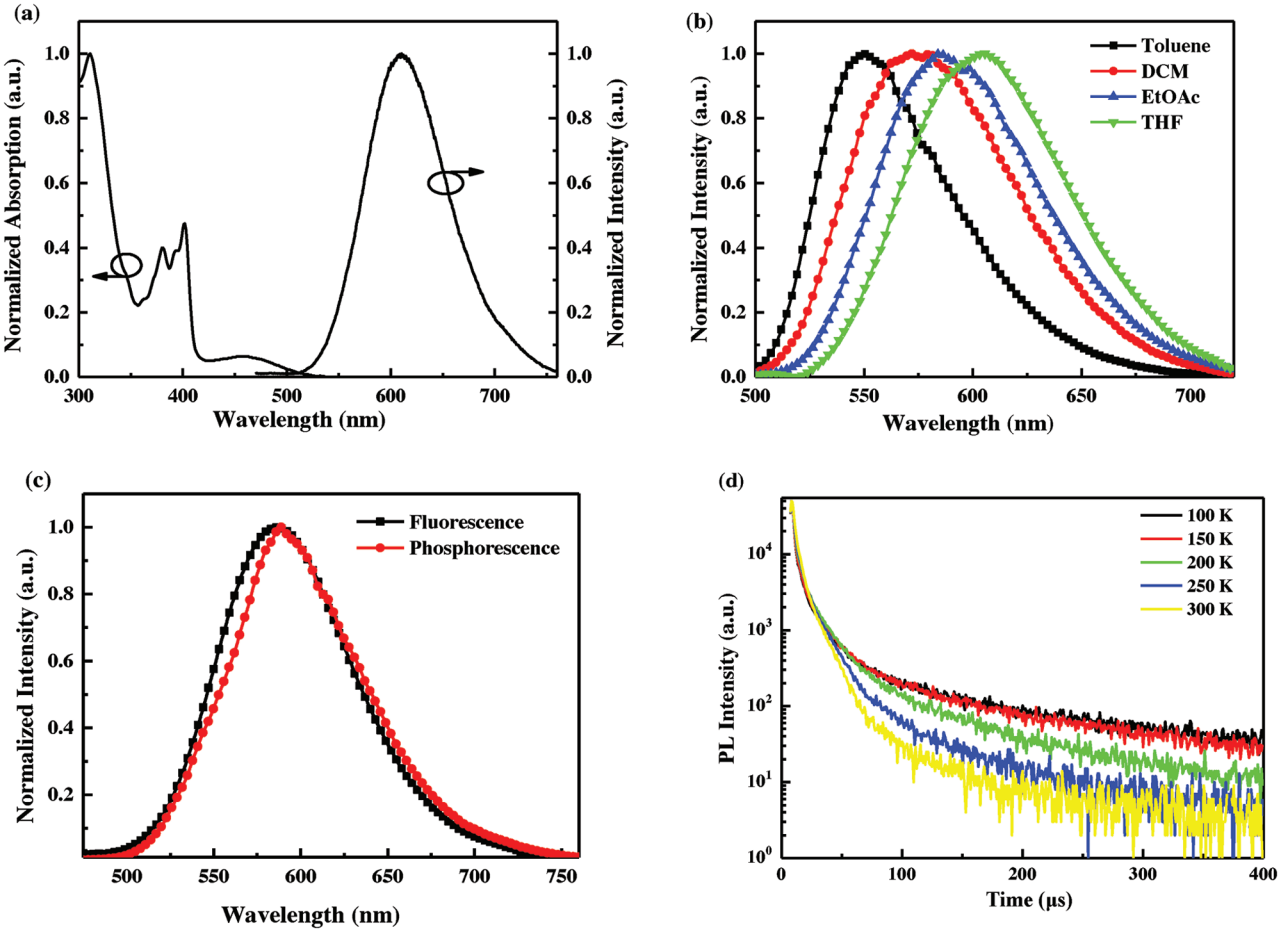
a) Normalized UV–vis absorption and emission spectra of DPXZ‐BPPZ in toluene at room temperature. b) The fluorescence spectra of in DPXZ‐BPPZ in toluene, DCM, EtOAc, and THF at 77 K. c) The fluorescence of DPXZ‐BPPZ 18 wt% doped CBP thin film at room temperature, its phosphorescence spectra at 77 K and d) transient PL decay curves at different temperatures.

Finally, we fabricated a multilayer OLED with an optimized structure of ITO/TAPC (35 nm)/TCTA (10 nm)/CBP:18 wt% DPXZ‐BPPZ (20 nm)/TmPyPb (45 nm)/LiF (1 nm)/Al. In the device, indium tin oxide (ITO) and Al were served as the anode and the cathode, respectively; TAPC (1,1‐Bis[4‐[*N,N*‐di(*p*‐tolyl)amino]phenyl]‐cyclohexane) and TmPyPb (1,3,5‐tri[(3‐pyridyl)phen‐3‐yl]benzene) were used as hole‐transporting layer and electron‐transporting layer, respectively; TCTA (4,4′,4″‐tris(carbazol‐9‐yl)triphenylamine) was employed as the exciton‐blocking layer; LiF was acted as electron injection layer. DPXZ‐BPPZ was doped into CBP host with an optimized doping concentration of 18 wt% as the emitting layer.

As shown in **Figure** **4**a, DPXZ‐BPPZ exhibits red emission with a peak at 612 nm and a Commission Internationale de L'Eclairage (CIE) coordinate of (0.60, 0.40) in the device, which meets the general requirement for red emission (λ_Max_ > 600 nm and the CIE coordinates of (*x* ≥ 0.60, *y* ≤ 0.40)).^26^ More importantly, the device successfully achieves impressively high efficiencies. Maximum EQE of 20.1 ± 0.2%, maximum current efficiency (CE) of 30.2 ± 0.6 cd A^−1^ and maximum power efficiency (PE) of 30.9 ± 1.3 lm W^−1^ are achieved in forward‐viewing direction without any outcoupling optimization. Moreover, the efficiency roll‐off is also modest for DPXZ‐BPPZ‐based device. The EQEs remain 16.7 ± 0.3% with a luminance of 1000 cd m^−2^ and 12.2 ± 0.3% with a luminance of 5000 cd m^−2^, respectively. **Table** **1** summarizes the performance of the reported red OLEDs based on TADF mechanism. To the best of our knowledge, DPXZ‐BPPZ‐based device achieves the best performance as red TADF OLEDs up to now and reaches the theoretical efficiency limit (assuming the out‐coupling efficiency as 20%) for the first time.

**Figure 4:**
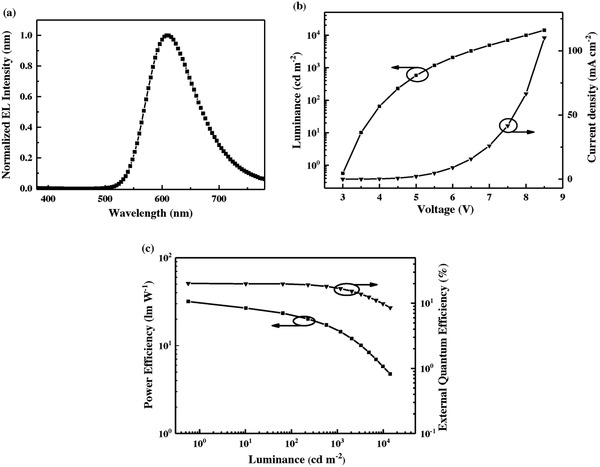
a) EL spectra of the device at 1000 cd m^−2^; b) current density–voltage–luminance curves; and c) PE–luminance–EQE curves of DPXZ‐BPPZ‐based device.

**Table 1:** Summary of performances of red TADF OLEDs (EL_max_ ≥ 610 nm)

Emitter	*V*_on_ [V]	λ_Max._ [nm]	CE/PE/EQE [cd A^‒1^/lm W^‒1^/%]		CIE (*x*, *y*)	Ref.
			Maximum	@1000 cd m^−2^		
DPXZ‐BPPZ	3.1	612	30.2 ± 0.6/30.9 ± 1.3/20.1 ± 0.2	25.5 ± 0.5/14.6 ± 0.6/16.7 ± 0.3	(0.60, 0.40)	This work
HAP‐3TPA	4.4	610	25.9/22.1/17.5	–/–/5.5a)	(0.60, 0.40)	27
AQ‐TPA	3.0	624	–/–/12.5	–/–/2.3	(0.61, 0.39)	17
b2	3.0	637	–/–/9.0	–/–/1.7	(0.63, 0.37)	17
POZ‐DBPHZ	3.7	≈610a)	–/–/≈16a)	–/–/–	(–, –)	15
TPA‐DCPP	3.1	668	4.0/–/9.8	–/–/2a)	(0.68, 0.32)	25
DPA‐Ph‐DCPP	3.2	644	13.2/12.9/15.1	–/3.5/1a)	(0.64, 0.36)	26
DPA‐DCPP	3.0	616	14.4/15.1/10.4	–/0.2/0.8a)	(0.61, 0.38)	26
DMAC‐DCPP	3.3	624	12.8/12.2/10.1	–/2/3a)	(0.60, 0.40)	26
APDC‐DTPA	–	693	–/–/10.19	–/–/–	(–, –)	33

^a)^ Estimated from the graphs in the references.

In conclusion, we have designed and synthesized a novel red TADF emitter DPXZ‐BPPZ. Due to the rigid and aromatic planar constituent groups and large steric hindrance between D and A segments, DPXZ‐BPPZ simultaneously achieves an efficient fluorescent radiation transition (*k*_F_/*k*_IC_ ≈ 33) and an effective RISC process (*k*_RISC_/$k_{\mathrm{nr}}^{T}$ = 44.8), thereby possesses an extremely high *Φ*_PL_ of 97.1 ± 1.1% under oxygen‐free condition. In the device, DPXZ‐BPPZ shows red emission with a peak at 612 nm and a CIE coordinate of (0.60, 0.40), and obtains high maximum forward‐viewing efficiencies of 20.1 ± 0.2% EQE, 30.2 ± 0.6 cd A^−1^ CE, and 30.9 ± 1.3 lm W^−1^ PE. This is the best performance among the reported red TADF OLEDs. These results prove DPXZ‐BPPZ is an ideal candidate for red TADF emitters, and we also present a new approach for designing highly efficient red TADF emitters.

## Experimental Section

*General Information*: NMR spectra were recorded in CD_2_Cl_2_ via a Bruker Advance‐600 spectrometer. Mass spectra data were obtained using Trace‐ISQ gas chromatography (GC)‐MS instrument and Bruker autoflex MALDI‐TOF mass spectrometer. Theoretical calculations were performed using the Gaussian‐09 program. The structure was determined and optimized by using DFT B3LPY/6‐31G (d). TGA measurement was using TAQ 500 thermogravimeter by measuring the weight loss of the specimen while heating at nitrogen atmosphere with a rate of 10 °C min^−1^ from 20 to 700 °C. Absorption and PL spectra were recorded on a Hitachi UV–vis spectrophotometer U‐3900 and a Hitachi fluorescence spectrometer F‐4600, respectively. The PL quantum yield (*Φ*_PL_) and transient PL measurements were measured by an Edinburgh Instruments FLS980 spectrometer. Cyclic voltammetry was carried out on a CHI660E electrochemical analyzer using silver chloride electrode as the reference electrode, Pt disk as the working electrode at a scan rate of 0.05 V s^–1^ with 0.1 m tetrabutylammonium perchlorate as the supporting electrolyte. The potentials of silver chloride electrode in dimethylformamide were calibrated to be 4.62 V relative to the vacuum level.

*Materials*: All the organic materials used in this study were purchased from various sources and used as received.

*Synthesis of 3,6‐Di(10H‐Phenoxazin‐10‐yl)Phenanthrene‐9,10‐Dione (DPXz‐PhO)*: PXZ (0.8 g, 4.4 mmol), 3,6‐dibromophenanthrene‐9,10‐dione (0.72 g, 2 mmol), cesium carbonate (2.64 g, 8 mmol), tri‐*tert*‐butylphosphane in 10% toluene solution (1 mL, 0.3 mmol),and palladium (II) acetate (22.4 mg, 0.1 mmol) was added into a round‐bottom flask (100 mL) at nitrogen atmosphere, followed by adding 40 mL of dry toluene. Then, the mixture was stirred at 110 °C for 24 h. After completion of the reaction, water and DCM were added to the cooled mixture. The organic layer was separated, and dried over Mg_2_SO_4_, and concentrated in vacuo. The residue solid was purified by column chromatography to give the product as black solid (0.69 g, 60.5%): ^1^H NMR (600 MHz, CD_2_Cl_2_, δ) 8.42 (d, *J* = 8.2 Hz, 2H), 7.98 (s, 2H), 7.55 (d, *J* = 8.2 Hz, 2H), 6.75–6.70 (m, 8H), 6.65 (t, *J* = 7.4 Hz, 4H), 6.14 (d, *J* = 7.9 Hz, 4H). MS (EI) *m*/*z*: 570.19 [M]^+^ calcd for C_38_H_22_N_2_O_4_ 570.16.

*Synthesis of DPXZ‐BPPZ*: DPXZ‐PhO (0.57 g, 1 mmol) and 1,10‐phenanthroline‐5,6‐diamine (0.21 g, 1 mmol) were added into 10 mL of 1‐butanol and then refluxed overnight under nitrogen atmosphere. After completion of the reaction, the mixture was poured into water. The separated solid was filtered off, washed with water, and then dried under vacuum. The crude product was purified by column chromatography to give the product as red solid (0.48 g, 65%). ^1^H NMR (600 MHz, CD_2_Cl_2_, δ): 9.96 (d, *J* = 8.1 Hz, 2H), 9.88 (d, *J* = 8.4 Hz, 2H), 9.40 (s, 2H), 8.64 (d, *J* = 1.7 Hz, 2H), 8.01 (s, 2H), 7.90 (dd, *J* = 8.5, 1.8 Hz, 2H), 6.73 (dd, *J* = 8.0, 1.4 Hz, 4H), 6.68 (t, *J* = 7.1 Hz, 4H), 6.62–6.58 (m, 4H), 6.13–6.07 (m, 4H). ^13^C NMR measurement was not possible due to low solubility. MALDI‐TOF MS (mass *m*/*z*): 744.33 [M]^+^; calcd for C_50_H_28_N_6_O_2_: 744.73.

*Device Fabrication*: ITO‐coated glass substrates with a sheet resistance of 15 Ω per square were cleaned with ethanol and deionized water, followed by drying in an oven at 100 °C, then subjected to UV–ozone treatment for 15 min, and finally transferred to a vacuum deposition system with a base pressure of approximately 4 × 10^–4^Pa. The organic layers were deposited on the ITO substrates with an evaporation rate of 1–2 Å s^–1^. The cathode layer was completed via the thermal deposition of LiF and Al at a rate of 0.1 and 10 Å s^−1^, respectively. EL luminance, CIE color coordinates, and spectra were recorded using a Spectrascan PR655 photometer, and the current density–voltage–luminance characteristics of the devices were measured with a computer‐controlled Keithley 2400 Source Meter under ambient atmosphere. EQE was calculated from the current density, luminance, and EL spectrum, assuming a Lambertian distribution.

## Conflict of Interest

The authors declare no conflict of interest.

## Supporting information

Supplementary
