# Supplementary material for: Red Organic Light‐Emitting Diode with External Quantum Efficiency beyond 20% Based on a Novel Thermally Activated Delayed Fluorescence Emitter
Source: Adv Sci (Weinh). 2018 Jul 20;5(9):1800436. doi: 10.1002/advs.201800436 (PMC6145404; doi:10.1002/advs.201800436)
Supplement: Supplementary file 1 — Supplementary [file ADVS-5-1800436-s001.pdf]

Copyright WILEY-VCH Verlag GmbH & Co. KGaA, 69469 Weinheim, Germany, 2018.

ADVANCED SCIENCE
Open Access

Supporting Information

for *Adv. Sci.,* DOI: 10.1002/advs.201800436

Red Organic Light-Emitting Diode with External Quantum Efficiency beyond 20% Based on a Novel Thermally Activated Delayed Fluorescence Emitter

Jia-Xiong Chen, Kai Wang, Cai-Jun Zheng,* Ming Zhang, Yi-Zhong Shi, Si-Lu Tao, Hui Lin, Wei Liu, Wen-Wen Tao, Xue-Mei Ou, and Xiao-Hong Zhang**

Copyright WILEY-VCH Verlag GmbH & Co. KGaA, 69469 Weinheim, Germany, 2016.

Supporting Information

Red Organic Light Emitting Diode with External Quantum Efficiency beyond 20% Based on a Novel Thermally Activated Delayed Fluorescence Emitter

Jia-Xiong Chen, Kai Wang, Cai-Jun Zheng,* Ming Zhang, Yi-Zhong Shi, Si-Lu Tao, Hui Lin, Wei Liu, Wen-Wen Tao, Xue-Mei Ou, and Xiao-Hong Zhang**

Supplementary Figures and Table

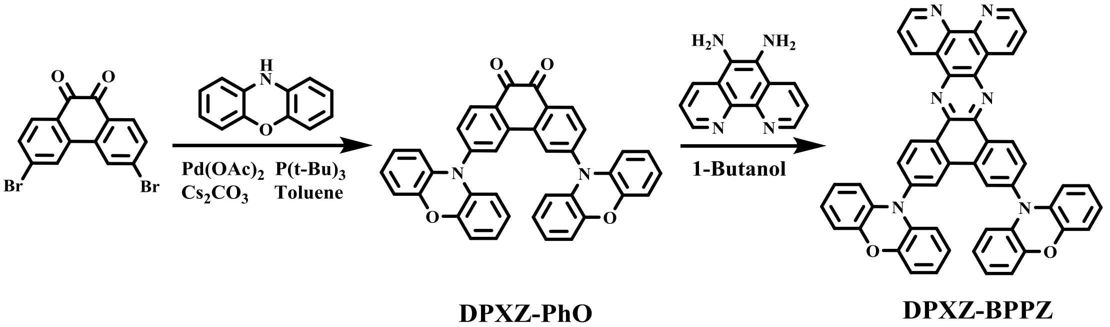

Scheme S1. Synthetic routes of DPXZ-BPPZ

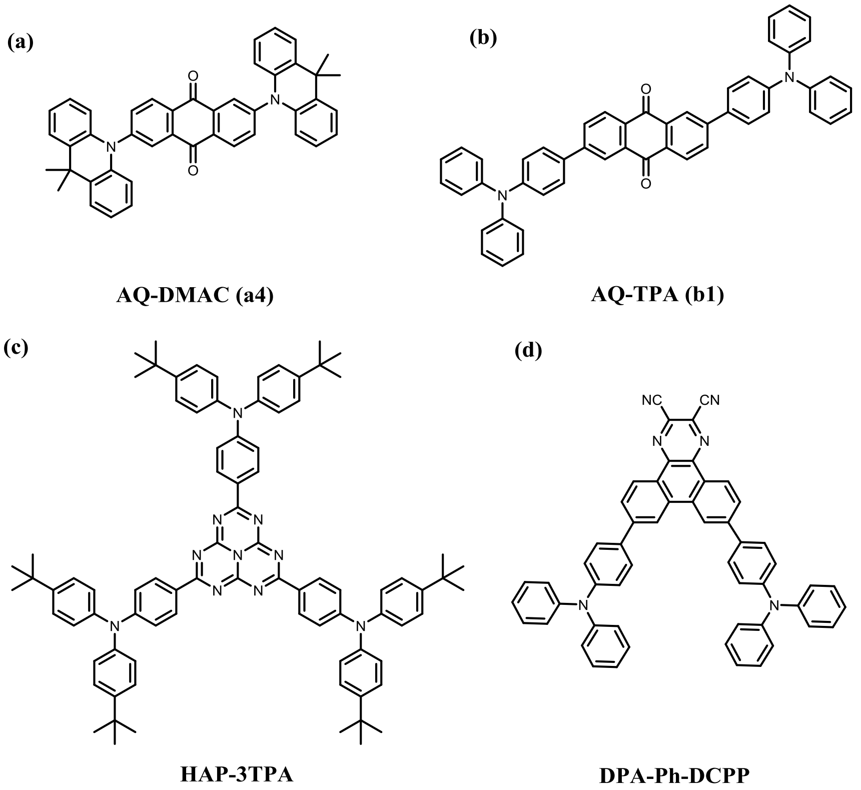

Figure S1. The structures of AQ-DMAC (a4), AQ-TPA (b1), HAP-3TPA and DPA-Ph-DCPP.

Table S1. Summary of physical properties of DPXZ-BPPZ.

Compound	$\lambda_{flou.}$ [nm][a)]	S_1 [eV][b)]	T_1 [eV][c)]	ΔE_{ST} [eV][d)]	Φ_{PL} [%]	T_g/T_d [°C]
DPXZ-BPPZ	610	2.41	2.38	0.03	97.1 ± 1.1	n.a./482

[a)]Determined from the emission peak of 10^{-5} M toluene solution at room temperature; [b)] Estimated from the onset of fluorescence spectrum; [c)] Estimated from the onset of phosphorescence spectrum at 77 K; [d)] $\Delta E_{ST} = S_1 - T_1$.

Table S2. Summary of Photophysical Parameters for Red TADF emitters.

Emitter	Φ_{PL} [%]	k_F [$\times 10^7$ s^{-1}]	τ_F [ns]	τ_{TADF} [μs]	k_{IC} [$\times 10^7$ s^{-1}]	k_{ISC} [$\times 10^7$ s^{-1}]	k_{TADF} [$\times 10^4$ s^{-1}]	Ref.
DPXZ-BPPZ	97	1.18	33	10.3	< 0.036	1.85	9.23	This work
AQ-DMAC	8	0.3	16.5	1.6	3.5	2.3	5.1	17
AQ-TPA	80	5.3	10.2	416	1.3	3.2	0.19	17
b2	76	5.3	10.2	185	1.7	2.8	0.41	17
HAP-3TPA	91	27	3.3	48.0	-	-	-	27
DPA-Ph-DCPP	49	2.4	20.1	82.1	2.0	0.50	0.73	26
DPA-DCPP	59	4.4	13.4	579	2.1	0.54	0.1	26
DMAC-DCPP	33	0.15	53.7	2.4	0.3	1.4	13.7	26

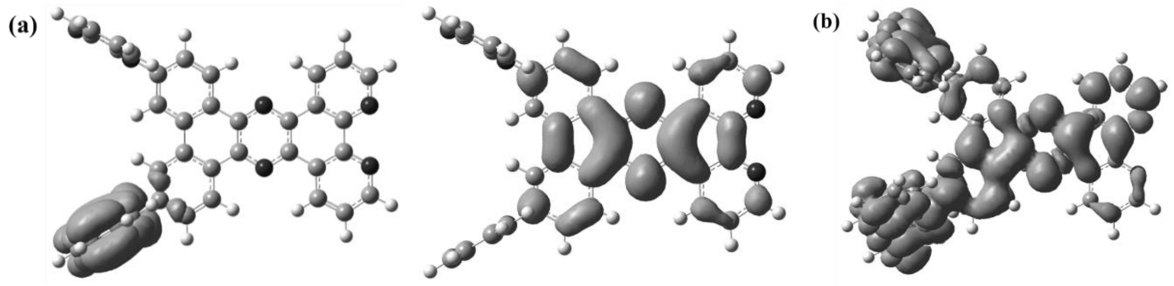

Figure S2. (a) The natural transition orbitals (NTOs) of lowest excited singlet states and (b) calculated spin-density distributions of the lowest-excited triplet states for DPXZ-BPPZ.

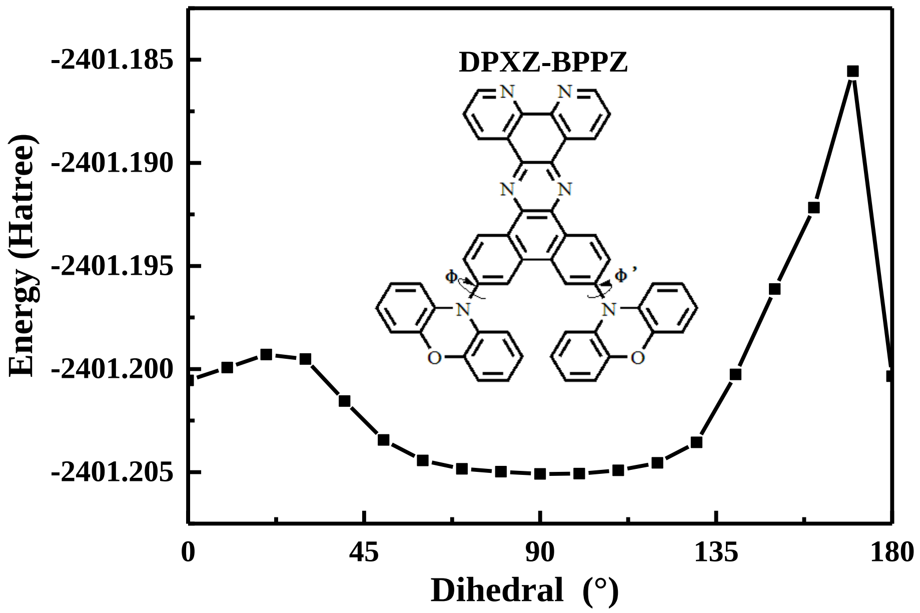

Figure S3. Potential energy surface of ground state of DPXZ-BPPZ in vacuum, φ and φ′ represent the dihedrals between PXZ and DPPZ, which, φ′, is optimized and fixed as considered normal nearly orthogonal conformations to simplify variables.

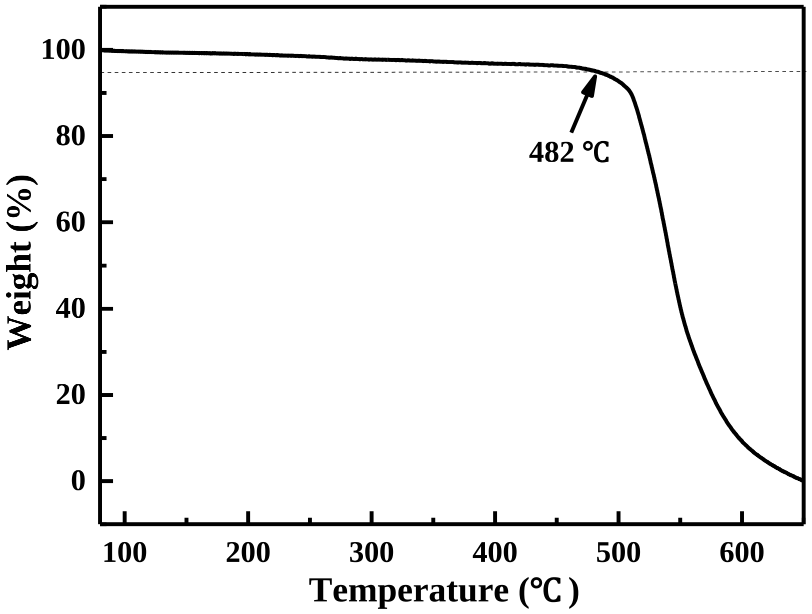

Figure S4. TGA measurement of DPXZ-BPPZ.

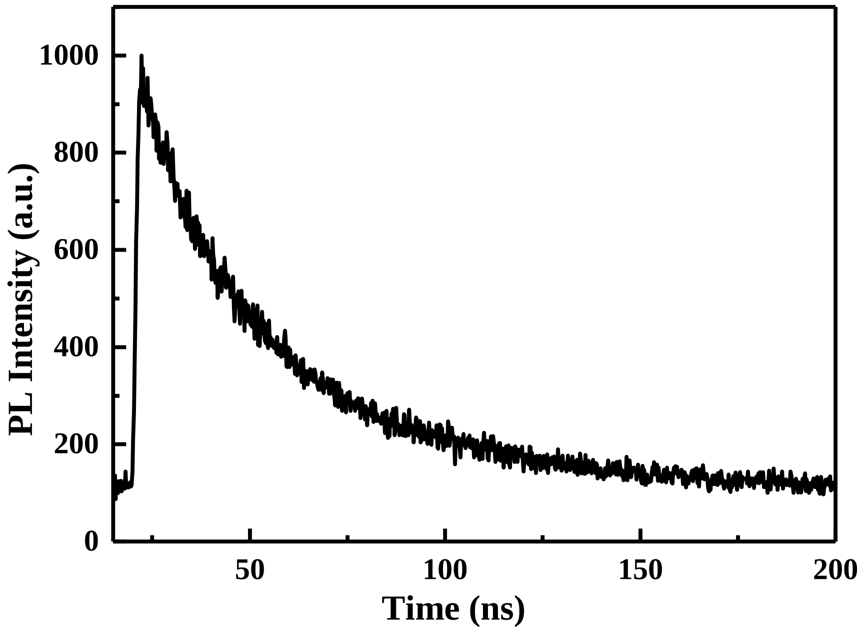

Figure S5. Transient PL decay curves of DPXZ-BPPZ at room temperature in the range of 200 ns.

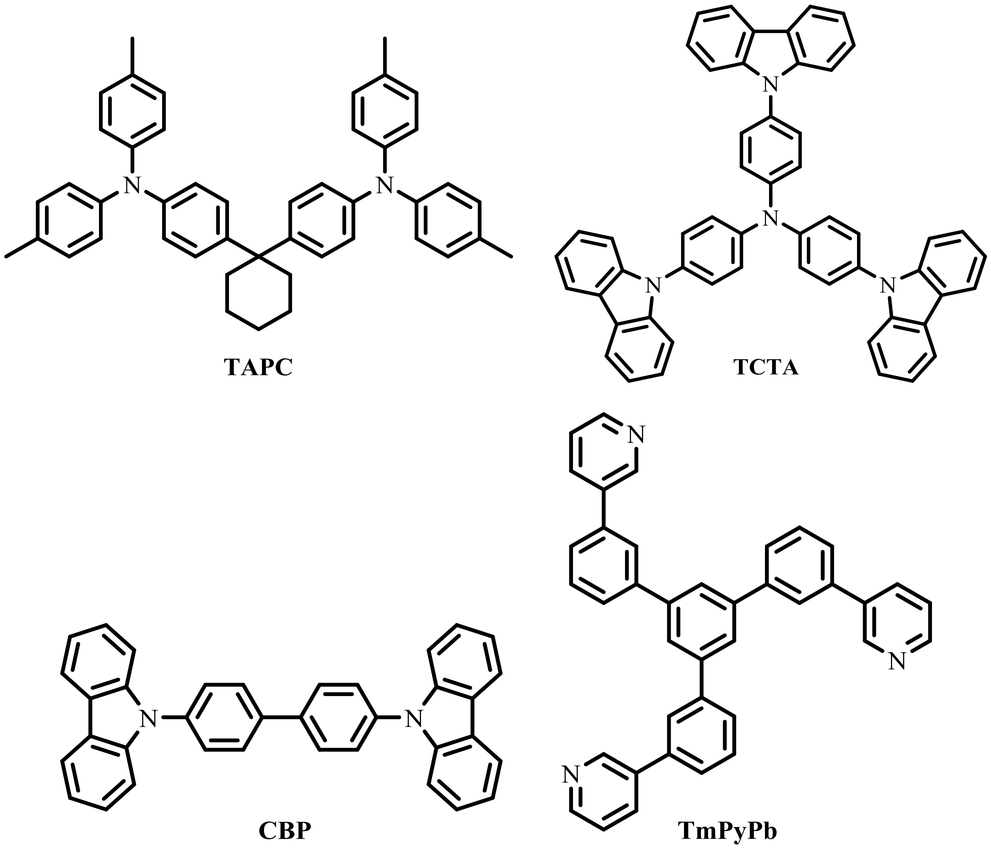

Figure S6. Molecular structures of TAPC, TCTA, CBP and TmPyPb.
